# Why are there deadly drugs?

**DOI:** 10.1186/s12916-015-0270-2

**Published:** 2015-02-05

**Authors:** Joel Lexchin

**Affiliations:** School of Health Policy and Management, York University, 4700 Keele St., Toronto, Ontario M3J 1P3 Canada; University Health Network, Toronto, Ontario M5G 2C4 Canada; Department of Family and Community Medicine, University of Toronto, Toronto, Ontario M5G 1V7 Canada

**Keywords:** Adverse drug reactions, Clinical trials, Drug regulatory authority, Drug safety, Pharmaceutical company

## Abstract

Some drugs eventually have to be removed from the market because of a negative benefit-to-harm ratio, including an excess of mortality. Drug safety is the result of multiple factors, commencing with how clinical trials are designed, the information generated by and/or hidden through these trials, trial analysis by drug regulatory authorities (DRAs) and the amount of information that DRAs choose to release, the amount of published information regarding drug safety, the effectiveness of postmarket surveillance systems in recognizing and reporting adverse drug reactions, and the structure of DRAs such as the United States Food and Drug Administration and its equivalent in other countries. This commentary will look at each of these issues in order to highlight the problems in the current approach to drug safety and finally indicate how some of these deficiencies should be addressed.

Please see related article: http://dx.doi.org/10.1186/s12916-014-0262-7

All drugs will cause death in some people at some point, but only a very few drugs are removed from the market for this reason. In a research article published in *BMC Medicine*, Onakpoya and colleagues [1] provide the first systematic examination of drugs withdrawn from the market because of a link to mortality. Their study highlights a number of issues that bear further examination. Why do so few withdrawals occur in African countries as opposed to European ones and why are many drugs not withdrawn worldwide? Second, they find that we are getting better at recognizing drug-related mortality but no better at dealing with the problem. While we wait to take action on these drugs, millions of people are being exposed to potentially deadly medications [2]. Although the article deals with drugs that lead to death at both therapeutic doses and overdoses, this commentary only deals with the former group.

## Where do withdrawals occur?

The question of where withdrawals occur points to a problem that has long been recognized; only a few developing countries have a well-functioning drug regulatory authority (DRA) [3] and pharmacovigilance systems in these countries are generally rudimentary [4]. Dealing with these problems is something that the global community needs to tackle, but the lead agency in this regard, the World Health Organization, is affected by a lack of resources [5].

## Factors involved in recognizing and dealing with potentially unsafe drugs

The finding that we are recognizing problems more quickly but failing to deal with them any faster suggests that some elements in the drug safety system are improving whereas others are not seeing any progress. Drug safety is the result of multiple factors starting with how clinical trials are designed, what information these trials generate and what may be hidden, how the trials are analyzed by DRAs, what is published and what is not, how well the postmarket surveillance system functions, and the structure of DRAs such as the United States Food and Drug Administration (FDA) and its equivalent in other countries.

## Recognizing dangerous drugs in clinical trials

It is well known that clinical trials are primarily designed to show that a drug is efficacious and not to detect anything other than relatively common safety problems. Who is included and excluded from clinical trials is important. If certain groups of people are not included then, when doctors prescribe for them, they have little or no information about what kind of side effects may occur. That typically means a lack of knowledge of how the drug will behave in people who are taking other medications, who have other chronic conditions, or who may not metabolize the drug in a well understood manner, i.e., children or the elderly. Clinical trials typically enroll, at most, about 5,000 patients and, therefore, any side effect, serious or trivial, that occurs in fewer than about 1 in 1,700 people will not necessarily be observed.

There have been a number of documented instances where pharmaceutical companies failed to provide mortality data to the FDA in a timely manner minimizing the appearance of a mortality risk and producing an apparent decrease in the danger associated with the drug [6,7].

Are reviewers within DRAs picking up on safety signals in clinical trials? In most of the world the answer is that we do not know. Drug safety reports are publicly available on the FDA website, but are redacted to remove confidential information. Woloshin and Schwartz have called on the FDA to make information more accessible by creating “*standardized executive summaries of drug reviews that quantify the benefit and important harms found in the phase III trials*” and that highlight remaining uncertainties [8]. Documents from some regulatory agencies that are supposed to outline the clinical basis behind the approval of new drugs do not disclose all the relevant information necessary to adequately assess the safety of the products [9,10]. Speeding up reviews of new drug submissions may get new drugs onto the market faster, but at the expense of more safety problems once they are being used by patients [11,12].

## Under-reporting of drug safety information

Safety information is routinely under-reported in published reports of randomized clinical trials. In one assessment of publications in six high-impact general medical journals, no information on severe adverse events and withdrawal of patients owing to an adverse event was given in 27.1% and 47.4% of articles, respectively. Restrictions in the reporting of harm-related data appeared in under one-third of articles [13]. Similar findings have been reported in other specialty journals [14].

There is significant under-reporting of adverse drug reactions. A systematic review of 37 studies from 12 countries found that the median under-reporting rate was 94% [15]. Even for extremely serious reactions, such as toxic epidermal necrolysis, under-reporting may be as high as 96% [16]. When reports contain insufficient detail, then doctors (and patients) have a false sense of security about the safety of the products that they are prescribing and using. The number of serious and fatal adverse drug reactions reported to the FDA between 1998 and 2005 went up 2.6- and 2.7- fold, respectively; reported serious events increased four times faster than the total number of outpatient prescriptions [17].

Complicating under-reporting are documented incidents where drug companies have disguised the nature of the reactions that they have reported to DRAs. Primary pulmonary hypertension was a recognized problem with dexfenfluramine and therefore reports about this condition were expected, whereas heart valve damage was unknown and reports about this side effect were unexpected [18]. An analysis of adverse drug event reports from the pharmaceutical company involved showed that many reports listed primary pulmonary hypertension first and heart valve damage second possibly in order to downplay the discovery of this new and significant problem [18].

## The role of regulatory agencies in handling adverse drug reactions

Many DRAs prioritize the drug approval process over drug safety. Within one agency, over three times the money and three times the number of personnel are devoted to the former as opposed to the latter [19]. In another agency, the hierarchical relationship between the office that approves new drugs and the one that monitors postmarket safety elevates the opinions of officials in the former above those of the epidemiologists, drug safety specialists, and risk communications officers who evaluate safety [20].

Once adverse drug reactions are recognized, they may still not be communicated appropriately and in a timely manner. For example, the FDA wanted to add a warning to the rofecoxib label about cardiovascular risks in light of the findings from the VIGOR trial, but there were objections from the pharmaceutical industry. The resulting negotiations took over a year to ultimately lead to a change and rather than going into the “warning” section of the label, it ended up in the less prominent “precautions” section and was said to be of unknown clinical significance [8].

## Conclusions

What is improving and what is not in this complex interplay of factors? The bottom line is that we have not collected the data to assess this. The first step in decreasing the number of people who die from drugs is to understand which issues to focus on. To that end, the first steps involve better resourcing of the postmarket surveillance system, placing drug safety on a par with drug approvals, and increasing the transparency of information from both pharmaceutical companies and DRAs.

## Abbreviations

DRA: Drug regulatory authority
FDA: United States Food and Drug Administration

## References

[1] Onakpoya I, Heneghan C, Aronson J. Delays in the post-marketing withdrawal of drugs to which deaths have been attributed: a systematic investigation and analysis. BMC Medicine 2015. 10.1186/s12916-014-0262-7 PMC4318389 25651859

[2] Friedman M, Woodcock J, Lumpkin M, Shuren J, Hass A, Thompson L (1999). The safety of newly approved medicines: do recent market removals mean there is a problem?. JAMA.

[3] World Health Organization (2004). The world medicines situation report.

[4] Pal S, Dodoo A, Mantel A, Olsson S (2011). The world medicines situation 2011: pharmacovigilance and safety of medicines.

[5] Kamal-Yanni M, Saunders P. Urgent need for WHO’s reform to prioritise core functions. Lancet. 2012;379:1878. 10.1016/S0140-6736(12)60810-1 22608337

[6] Lurie P, Wofle SM (2005). Misleading data analyses in salmeterol (SMART) study. Lancet.

[7] Psaty BM, Kronmal RA (2008). Reporting mortality findings in trials of rofecoxib for Alzheimer disease or cognitive impairment: a case study based on documents from rofecoxib litigation. JAMA.

[8] Woloshin S, Schwartz L (2009). Bringing the FDA’s information to market. Arch Intern Med.

[9] Habibi R, Lexchin J. Quality and quantity of information in summary basis of decision documents issued by health Canada. PLoS One. 2014;9:e92038. 10.1371/journal.pone.0092038 PMC3961288 24651766

[10] Barbui C, Baschirotto C, Cipriani A. EMA must improve the quality of its clinical trial reports. BMJ. 2011;342:d2291. 10.1136/bmj.d2291 21613341

[11] Carpenter D, Zucker E, Avorn J (2008). Drug-review deadlines and safety problems. New Engl J Med.

[12] Lexchin J (2012). New drugs and safety: what happened to new active substances approved in Canada between 1995 and 2010?. Arch Intern Med.

[13] Pitrou I, Boutron I, Ahmad N, Ravaud P (2009). Reporting of safety results in published reports of randomized controlled trials. Arch Intern Med.

[14] Breau R, Gaboury I, Scales C, Fesperman S, Watterson J, Dahm P (2010). Reporting of harm in randomized controlled trials published in the urological literature. J Urol.

[15] Hazell L, Shakir S (2006). Under-reporting of adverse drug reactions: a systematic review. Drug Saf.

[16] Mittmann N, Knowles S, Gomez M, Fish J, Cartotto R, Shear N (2004). Evaluation of the extent of under-reporting of serious adverse drug reactions: the case of toxic epidermal necrolysis. Drug Saf.

[17] Moore T, Cohen M, Furberg C (2007). Serious adverse drug events reported to the Food and Drug Administration, 1998–2005. Arch Intern Med.

[18] Mundy A (2001). Dispensing with the truth: the victims, the drug companies, and the dramatic story behind the battle over fen-phen.

[19] Wiktorowicz M, Lexchin J, Moscou K, Silversides A, Eggertson L (2010). Keeping an eye on prescription drugs, keeping Canadians safe: active monitoring systems for drug safety and effectiveness in Canada and internationally.

[20] Carpenter D (2010). Reputation and power: organizational image and pharmaceutical regulation at the FDA.

